# The factors associated with being left-behind children in China: Multilevel analysis with nationally representative data

**DOI:** 10.1371/journal.pone.0224205

**Published:** 2019-11-11

**Authors:** Lian Tong, Qiong Yan, Ichiro Kawachi

**Affiliations:** 1 Department of Maternal and Child Health, School of Public Health, Fudan University, Shanghai, China; 2 Department of Social and Behavioral Sciences, Harvard T.H. Chan School of Public Health, Boston, United States of America; Institute of Economic Growth, INDIA

## Abstract

There are 69.7 million left-behind children (LBC) in China. Using nationally representative monitoring data for migrant workers aged 15 to 59 years in China, this study sought to estimate the prevalence of LBC in each province, and to examine risk factors being left behind at both the individual and provincial level. Data on a total of 117,573 children less than 18 years of age were included in the multilevel analysis. At individual and family level, children’s gender, age, family income, migration distance, parental separation, and housing condition in host cities were associated with being LBC. The average household monthly income in the host province was significantly associated with the migrant parents’ arrangement to leave their children behind. Comprehensive supportive policies and strategies on schooling, housing for the migrant family in host cities might be beneficial to reduce the number of LBC in China.

## Introduction

Since the economic reforms of the late 1970s, China has been experiencing the largest internal migration in human history, with hundreds of millions of migrants streaming into major cities each year. A report released in 2013 estimated that there were 105.5 million children who belong to migrant families, accounting for roughly a third of the total population of children aged below 18 years in China [1]. Migrant parents from rural areas typically decided to leave their children behind, creating a potentially vulnerable subpopulation of left-behind children (LBC) in both rural and urban areas. While a precise population count of LBC is not available, it has been estimated that 61.0 million LBC are living in villages with at least one parent working away as a migrant [1–2]. LBC generally refer to children who remain in rural regions of China while their parents migrant to work in urban areas, although some literature also included LBC in urban areas due to parents’ migration between cities (e.g., from smaller cities to major urban centers). The estimated number of urban LBC is 8.7 million, which account for 12.5% of whole LBC in China [2]. At the same time, many migrant parents did take their children (or some of their children) with them to the urban areas, resulting in 35.8 million migrant children (MC) living in cities. In 2013, about 63% of 6–15 years old rural children migrated together with their parents [3].

Parental migration has potentially long lasting adverse effects on children [4]. Both LBC and MC have been documented with significantly less favorable functioning across emotional, social, and academic domains compared to other Chinese children [5]. Various mental health problems have been identified among rural and urban LBC [6–8]. The psychosocial well-being and development of LBC is particularly threatened by the absence of parental affection, support and supervision [9]. In addition, LBC especially those living in low-income situations have an increased risk of committing juvenile crimes [10]. LBC are also more likely to engage in high risk behaviors, such as smoking, alcohol abuse, teenage pregnancy, as well as sexual and physical violence [11].

While these may be some benefits for children to migrate with their parents, migration into cities has a more complex impact on children’s health and development. MC may benefit from the excellent resources in cities; but they may also suffer from adverse living conditions and various forms of social stigma and discrimination [12]. With their marginalized status in cities, most migrants earning a minimum wage are not covered by health insurance, and they subsist in poor housing conditions. MC exhibited more problems in emotional well-being, as well as social and academic functioning compared to local urban children [13]. However, with the Household Registration System (hukou) and education system reforms, urban life for MC has improved over time [14]. They are more able to access public education and health care in the city [15]. Even though MC must confront the challenges of living in a new urban environment, their internal family structure remains largely intact [16]. Limited studies compared the health and developmental outcomes between LBC and MC, and suggested that MC have better nutritional status, physical and mental health compared to LBC [17, 18]. MC were better able to access social & educational resources, as well as infrastructure & services, compared to LBC [19]. Therefore an important question is why many parents chose to leave their children behind and what the factors that are associated with such decisions or arrangements.

Many existing studies have explored the reasons for the growing LBC populations in China, but they were rarely guided by a conceptual framework. Ecological theory as one of the system theory suggested that children do not develop in isolation, but within a system of relationships that include family and society [20,21]. The ecological theory is especially appropriate to study the causes for LBC from a holistic perspective, because LBC are the results of a large social context of rapid urbanization in China in recent three decades. There are both macrosystem factors and microsystem factors that may have contributed to phenomena of LBC. First, some institutional factors in the macrosystem level in Chinese society have posed barriers for migrant parents to bring their children to the destination cities. The mostly mentioned factor is the China’s social system based on the dual Household Registration System (hukou) implemented since the late 1950s. Generally, hukou is divided into agricultural and non-agricultural residency status (often referred to as rural and urban), which is assigned to different benefits from social programs provided by the government [22]. It has been used to deny temporary migrants access to the health care, education and welfare systems at the host cities. This meant that migrant families did not enjoy the same rights of access to local health care services, health insurance, prenatal care, and public education for their children compared to those residents with city hukou [23–26]. The hukou system is also tightly bound with housing policy. Migrants are not entitled to subsidize housing and other social welfare, and most are unable to buy housing due to rising retail house prices. Migrants are forced to rent through the private market, and live in low quality self-built housing, factory dorms, or even illegal housing, such as bomb shelters and storage basements [27, 28]. In addition to extreme crowding and poor housing conditions, migrants experience frequent residential relocations due to job insecurity (e.g. in the service and construction sectors) and demolition of their informal settlements. Thus, it can be very difficult for them to provide decent and stable shelters for their children in cities.

Family and individual factors in microsystem level may also contribute to the LBC phenomena, as they often directly affect children’ s living arrangement and living conditions. As existing empirical research exploring the causes of left behind children has mostly focused on social context, the family’s own characteristics were often overlooked. The family socio-economic status, migrated distance, the gender and age of children may all determine the family’ s decision to leave their children in hometown or bring them to cities. In family systems theory, families are seen as dynamic wholes. This perspective emphasizes the multifaceted roles played by all family members, including children themselves [29]. The macrosystem factors and microsystem factors are often interacting to affect the family’s decisions. For example, the parents’ living conditions in host cities may affect family arrangement. Therefore, research should not only pay attention to the factors in different ecological levels, but also examine the potential interactive effects of these factors in resulting LBC phenomena.

In conclusion, there are several knowledge gaps in the existing studies in studying LBC. First, there is a dearth of empirical research regarding the reasons for migrant parents to leave their children behind. Most of current discussions are drawn from subjective reasoning, rather than a quantitative analysis. Second, previous studies have mainly focused on institutional barriers, such as hukou and education system, but few studies have also examined the characteristics of children and migrant families including their socio-economic status, migration distance, as well as health care accessibility. Third, most previous studies have overlooked the factors at migration destinations. In a vast country like China, the economic developmental level, number of migrant workers, child education policy for migrant children, and housing purchase policies vary substantially across provinces as well as migration destinations and could affect the LBC phenomena. To address these knowledge gaps, the current study sought to apply multilevel analysis to examine individual and province level correlates of LBC in comparison with MC using data from a nationally representative migrant worker household survey.

## Methods

### Sampling procedure

The data utilized in this study were obtained from the surveillance survey of national health and family planning dynamic monitoring for migrant workers conducted by the National Health and Family Planning Commission of China in May 2014. The survey targeted those migrant households in urban areas. A household is eligible to participate if there was at least one migrant worker aged 15 to 59 years in the house. Migrants are defined as people who did not have a local hukou but lived in the host cities at least one month at the time of survey. The sampling method is stratified, multistage clustered probability and proportionate to size sampling (PPS). In total 200,937 households were recruited from 429 cities or urban districts in 32 provincial-level administrative units (provinces, autonomous regions, municipalities, and special administrative region) of mainland China. There were 11 to 27 cities or urban districts recruited from each provincial unit. Then, city street resident committees and village resident committees were selected from the qualified cities or urban districts by the method of PPS. All the migrated families administrated by the selected city street or rural village committees were divided into survey groups. Then, 100 qualified migrant families were selected from the survey groups. Finally, 20 families were selected depend on their gender, age and the time of migration.

**Fig 1:**
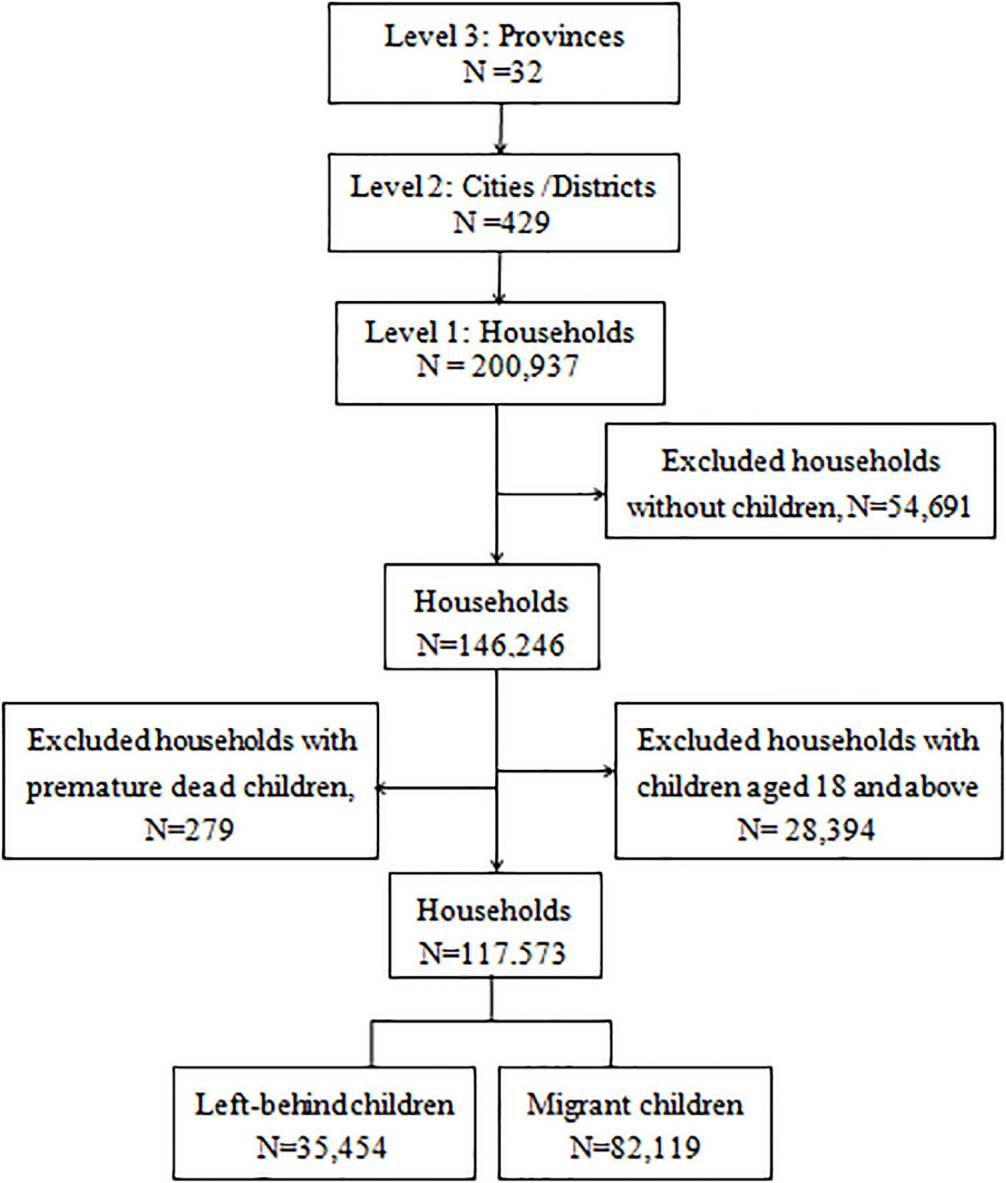
Schematic representation of the two-level hierarchical structure of the final analytic sample.

One adult aged 15–59 years was selected from each household to complete the survey. For participants who were parents, they were asked to provide detailed information on their children aged less than 18 years. If there were more than one child in that age range in the family, the first child that parents filled in the table was selected as the index child. Of the 200,937 participants, 54,691 (27.22%) reported having no children, 28,394 (14.13%) had only children aged over 18 years, and 279 (0.14%) reported having deceased children (Fig 1). After excluding those households, a final sample of 117,573 households nested within 429 cities or urban districts in 32 provinces or cities were included in this study.

### Ethical approval

The “National Internal Migrant Dynamic Monitoring Survey, 2014” data is publicly available to authorized researchers who have been given permission by the National Population and Family Planning Commission. Written informed consents were obtained from all participants. The analysis of public access data was exempted by the local IRB of Medical ethics committee of Fudan University. All data were provided in an anonymized format.

### Survey procedure

The questionnaire was developed by China Population and Development Research Center in Beijing, China. Local Population and Family Planning Commissions were responsible to recruiting and training the interviewers. The interviewers visited each migrant household and conducted face-to-face interviews with respondents privately at a predetermined time. A notification letter for participators and small gift were sent to their home before the official interview. If a selected participant withdraws, a new migrant household in the same area will be identified to recruit a person of same gender, similar age and migration time to the host city (as the withdrawing participant). Detailed information about demographics, employment history, income, housing, health, and social network, and information about their families including their children were collected during the survey. The research protocol including the recruitment procedures received administrative and ethnic review and approval by the National Bureau of Statistics of China.

### Measures

#### Children migratory status

Parents were asked where their children were living at the time of survey. This item was used to identify whether the child’s migratory status. Children who were living hometown and other places (but separated from their parents) were considered as LBC, while children who were living the host cities with their parents were considered as MC. Hence, the dependent variable is a binary response variable (whether a child is LBC or MC). All the following information in individual, family and provincial levels were considered as independent variables, which were put into models step by step.

#### Individual and family level factors

The parents were asked to provide information on children’s gender and age, parent’s education, parents’ hukou types (agriculture or non-agriculture), migration distance (across or within province), regions of migration destination (eastern, middle, western and northern areas), housing types (own or rent apartment, dormitory, live in workplace, etc.), and whether the parents live separately (yes or no). For the purpose of data analysis, children’s ages were divided into five groups in correspondence to the division of children’s age by the education system in China: ≤2, 3–5, 6–11, 12–14 and 15–17 years. Parent’s education levels were categorized into four groups: illiteracy and primary school, middle school, high school, and as least college.

#### Social welfare benefits

Participants were asked whether they were the recipients of three types of government’s social welfare benefits including the pension insurance for rural residents (yes or no), old-age insurance for urban workers (yes or no), or old-age insurance for urban residents (yes or no). Information was also collected on housing subsidies (yes or no), and unemployment insurance (yes or no).

#### Health insurance

Participants were asked whether they received any of the five common types of health insurance coverage available to Chinese residents: New Rural Cooperative Medical Insurance (yes or no), Basic Medical Insurance for Urban Workers (yes or no), Basic Medical Insurance for Urban Residents (yes or no), Cooperative Medical Insurance for Urban and Rural Residents (yes or no), and Maternity Insurance (yes or no).

#### Destination factors

Information regarding the migration destination included provincial factors such as GDP and family monthly income in the destination as well as the housing conditions in the destination. The GDP of each province were obtained for the report of National Bureau of Statistics of China. The families monthly incomes in the destination were obtained from participants’ self-report in this survey.

### Analysis

First of all, the chi-square test was used to examine differences between migrant families with LBC and MC with regard to the socio-demographic background of parents, housing, social welfare. Next, a multilevel modeling approach was applied to take full advantage of province clustering information. Thus, a three-level intercept random Poisson model was used to explore predictors at individual and province levels. The dependent variable is the proportion of LBC (LBC [1], and MC [0]). Each continuous variable was entered into the model after log transformation.

Multilevel model analysis was performed in a step-by-step manner. First, null model with only the intercept (model 1) was tested. Second, individual and family level covariates were entered (model 2). Third, provincial level explanatory variables were added to the model (model 3). All estimates were obtained by using 2nd-order predictive quasi-likelihood (PQL) to approximate linearization based on a Taylor series expansion to transfer discrete response to a continuous response model [30]. 2nd-order PQL is known to be more stable than the 1rt-order PQL or Marginal quasi-likelihood (MQL). Lastly, figures and maps were produced to visualize the geographic clustering of LBC and MC in destination provinces of China. The descriptive data analysis was conducted by Statistic Analysis System (SAS) version 9.3 (Institute Inc., Cary, NC, USA). Multilevel modeling was performed by MLwiN 2.36 version.

## Results

### Sample characteristics

Data from a total of 117, 573 children aged less than 18 years old were provided by their parents including 35,454 (30.1%) left-behind children and 82,119 (69.9%) migrant children. Among LBC, 31,203 (88%) of them had rural hukou, and these children were considered as rural LBC, while 4,251 (12%) children had urban hukou and were considered as urban LBC. Children’s average age is 8.6 years old (SD = 5.1). As shown in Table 1, about 53.3% of all children were boys, with a mean age of 8.7 years (SD = 5.1). The average age for girls was 8.4 years old (SD = 5.1). There were 36.1% children younger than five years, 33.7% children aged 6–12, and 30.2% children aged 12 to 17. A majority of the households (89.5%) had income between 300 to 1500 dollars per month. Most of the parents (70.8%) finished just middle school education or had an agriculture hukou (84.9%). Likewise, 84.4% of the children had an agriculture hukou.

**Table 1:** Differences between households with left-behind children and migrant children in socio-demographic characteristics, migrant status, housing, welfare, and health insurance.

	Left-behind children	Migrant children	Total
**Children’s gender**			
Boys	19162 (54.0)	43489 (53.0)	62651 (53.3)
Girls	16292 (46.0)	38630 (47.0)	54922 (46.7)
**Children’s age**			
0–2	4525(12.7)	16347 (19.9)	20872 (17.8)
3–5	5870 (16.5)	15674 (19.1)	21544 (18.3)
6–11	11347 (32.0)	28263 (34.4)	39610 (33.7)
12–14	6116 (17.3)	11647 (14.2)	17763 (15.1)
15–17	7596 (21.4)	10188 (12.4)	17784 (15.1)
Child number			
One	18988 (53.5)	50523 (61.4)	69511 (59.0)
Two	14421 (40.6)	28009 (34.0)	42430 (36.0)
Three and above	2075 (5.9)	3758 (4.6)	5833 (5.0)
**Parent’s age**			
<18	5 (0.01)	17 (0.02)	22 (0.02)
18–29	7499 (21.2)	19847 (24.2)	27346 (23.3)
30–39	16966 (47.9)	41711 (50.8)	58677 (49.9)
40–49	10529 (29.7)	19590 (23.9)	30119 (25.6)
50–59	455 (1.3)	954 (1.1)	1409 (1.2)
**Parent’s education**			
Illiteracy and primary school	5405 (15.2)	10491 (12.8)	15896 (13.5)
Middle school	20962 (59.1)	46502 (56.5)	67464 (57.3)
High school	6525 (18.4)	15676 (19.1)	22201 (18.8)
College and above	2592 (7.3)	9621 (11.7)	12213 (10.4)
**Household monthly income (USD)**			
<300	1702 (4.8)	2729 (3.3)	4431 (3.8)
300–750	18029 (50.8)	39916 (48.6)	57945 (49.3)
750–1500	14052 (39.6)	33160 (40.4)	47212 (40.1)
>1500	1671 (4.8)	6314 (8.7)	8985 (6.8)
**Parent’s hukou**			
Agriculture	31203 (88.0)	69707 (84.9)	100910 (85.9)
Non-Agriculture	4251 (12.0)	12412 (15.1)	16663 (14.1)
**Two parents live separately**			
Yes	10157 (28.6)	2299 (3.8)	12456 (10.6)
No	25297 (71.4)	79820 (97.2)	105177 (89.4)
**Migrant distance**			
Across provinces	22707(64.0)	37766 (46.0)	60473 (51.4)
Across cities within a province	7871 (22.2)	27259 (33.2)	35130 (29.9)
Across counties within a city	4876 (13.8)	17094 (20.8)	21970 (18.7)
**Migrant Region**			
Eastern areas	18686 (52.7)	32705 (39.9)	51391 (43.8)
Middle areas	5336 (15.1)	15637 (19.0)	20973 (17.8)
Western areas	10189 (28.7)	26540 (32.3)	36729 (31.2)
Northern areas	1243 (3.5)	7237 (8.8)	8480 (7.2)
**Housing**			
Rent a private apartment	24416 (68.9)	53970 (65.7)	78386 (66.7)
Own apartment	1993 (5.6)	20221 (24.6)	22214 (18.9)
Rent an apartment offered by employer	2175 (6.1)	2916 (3.5)	5091 (4.3)
Rent an apartment offered by government	104 (0.3)	337 (0.4)	441 (0.4)
Free dormitory offered by employer	5312 (15.0)	1840 (2.2)	7152 (6.1)
Live in workplace	999 (2.8)	1624 (2.0)	2623 (2.2)
Live in unstable place	454 (1.3)	1211 (1.5)	1665 (1.4)
**Social security**			
New type of pension insurance for rural resident)	19170 (54.1)	40912 (49.8)	57490 (48.9)
Old-age insurance for urban workers	6103 (17.2)	13926 (17.0)	20029 (17.0)
Old-age insurance for urban residents	1742 (4.5)	4742 (5.8)	6384 (5.4)
Housing subsidies	2119 (6.0)	5907 (7.5)	8026 (6.8)
Unemployment insurance	4668 (13.2)	10522 (12.8)	15190 (12.9)
**Health insurance**			
New Rural Cooperative Medical Insurance	24354 (68.7)	53895 (65.6)	78249 (66.6)
Basic Medical Insurance for Urban Workers	5676 (16.0)	13176 (16.0)	18852 (16.0)
Basic Medical Insurance for Urban Residents	1675 (4.7)	5373 (6.5)	7048 (5.6)
Cooperative Medical Insurance for Urban and Rural Residents	845 (2.4)	1643 (2.0)	2488 (2.1)
Maternity Insurance	3292 (9.3)	8493 (10.3)	11785 (10.0)
**Total**	35454 (30.1)	82119 (69.9)	117573(100.0)

### Demographic information of LBC and MC

As shown in Table 1, the proportions of left-behind children and migrant children were 30.1% and 69.9%, and slightly gender difference is displayed. There are 36.1% children aged 0 to 5 left behind. While 33.7% children aged 6 to 11 and 30.2% children aged 12 to 17 became LBC. There are 70.8% migrants only have middle school and below education. For 89.4% migrants, their household monthly income is between 300 to 1500 USD. There are 74.3% of migrant parents only finished middle school education or less. Overwhelmingly, 85.9% migrant parents and their children (85.4%) were identified as agriculture hukou. There are 28.6% LBC living with single parents, while only 3.8% of MC living with single parents.

### Patterns of parental migration & urban housing

On a national average, over half of migrant parents moved across provinces (51.4%), while the remainder migrated within provinces (48.6%). As expected across provinces migration yield more left-behind children (64.0%), comparing 36.0% for within provinces float (χ^2^ = 3232.6, p < 0.0001). Also, parents moved to the eastern areas of China accompanied with a high proportion of LBC (52.7%). Considering housing conditions, 66.7% migrant parents rent private apartments, and only 18.9% of them own apartments. There are 4.3% migrant parents who rent apartments offered by employers, and 6.1% of them live in free dormitories. Only 0.4% migrant parents rent an apartment provided by the government. A small group (3.6%) of households lived in the workplace or no stable dwelling. Most of the parents of left-behind children rent private apartments (68.9%), which is similar to the parents of migrant children (65.7). However, only 5.6% parents of LBC own their apartment, which is much lower than 24.6% for parents of MC. In contrast, 15.0% parents of LBC lived in free dormitories, which is higher than 2.2% for the parents of MC.

### Social welfare benefits and health insurance

With regard to endowment insurance, nearly half (48.9%) of migrant parents joined the new type of pension insurance for rural residents. 17.0% of them participated in the old-age insurance for urban workers. However, very few of them (6.8%) are qualified for housing subsidies. Obviously, most of the migrants remain excluded from the city welfare system. Access to the social welfare system appeared highly correlated with hukou system. Considering health insurance, more parents of MC joined medical insurance for urban residents and maternity insurance than parents of LBC (χ^2^ = 145.3, p < 0.0001; χ^2^ = 30.7, p < 0.0001). However, it should be noticed that the difference between two types of parents is subtle.

### Destination factors

**Fig 2:**
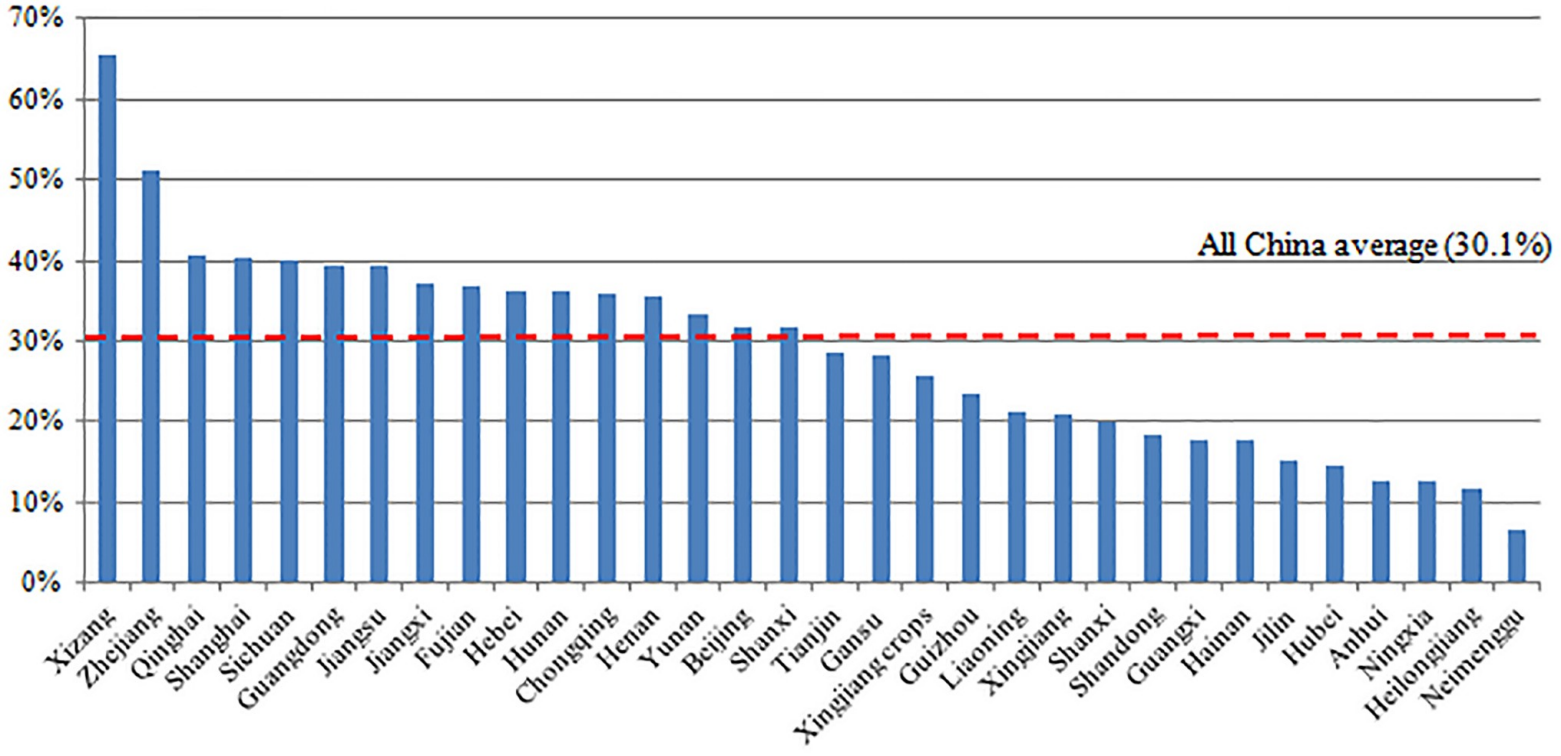
Percentage of left-behind children generated by migrant parent in 32 host provinces.

**Fig 3:**
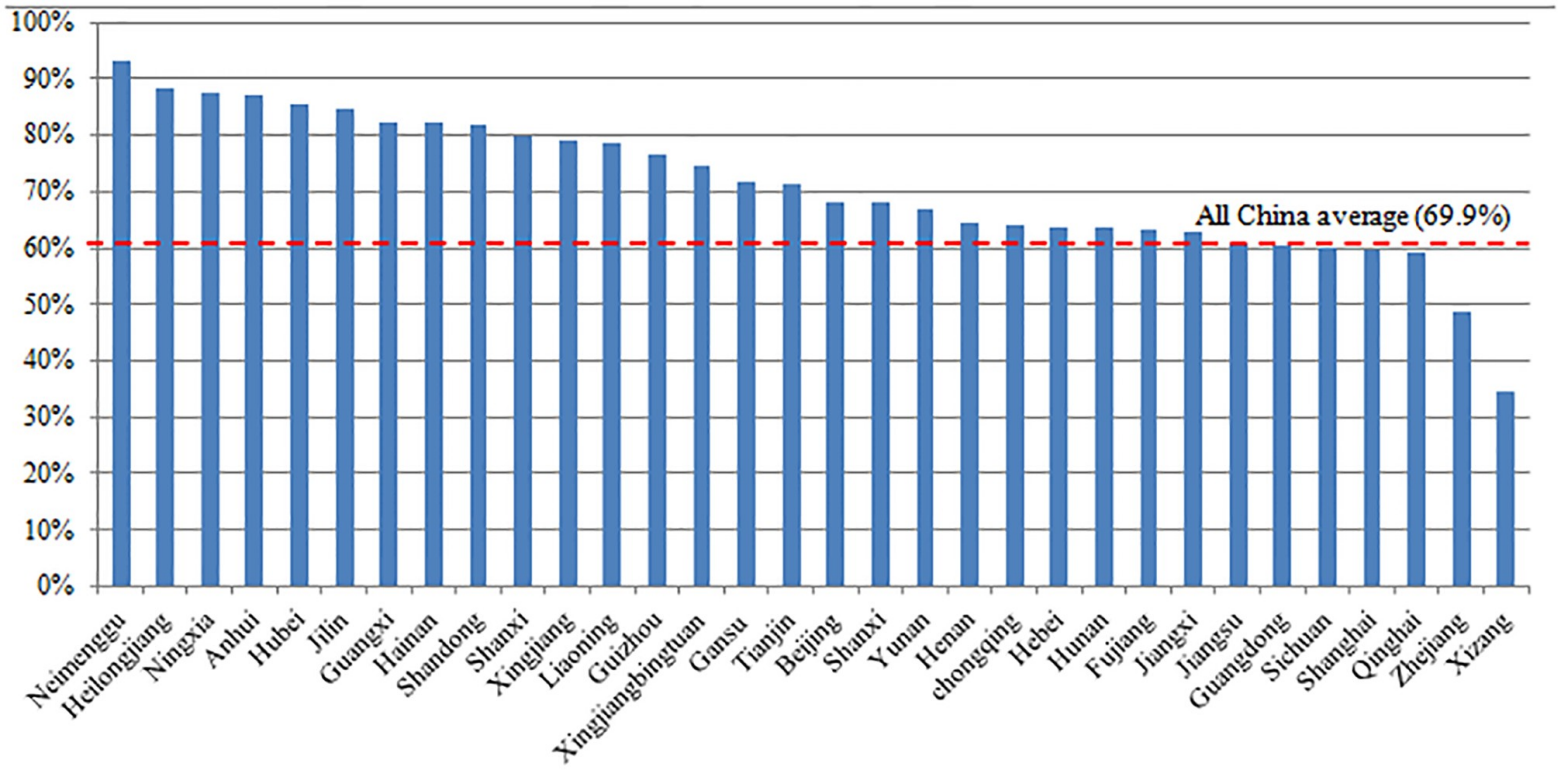
Percentage of migrant children in 32 host provinces.

The distribution of LBC and MC by destination (provinces) are shown in Table 2. It turns out that parents migrated to Xizang are most likely to leave their children behind (65.5%), followed by Zhejiang province (51.3%), Qinghai (40.8%) and Shanghai (40.2%) (Table 2, Fig 2). On the other hand, migrant parents worked in Neimenggu are most likely to bring children with them (93.3%), followed by Heilongjiang (88.4%), Ningxia (87.5%) and Anhui (87.2%) (Table 2, Fig 3). Even though on national average nearly half of migrant parents were across-province migrants, for some provinces the proportion of cross-province migrants can be extremely high, such as 100% for Beijing, Tianjin, and Shanghai, 97.1% for Xinjiang crops, 89.8% for Zhejiang. The averages of migrant household incomes vary by provinces. Meanwhile, a wide income inequality is shown within provinces as suggested by the high standard deviation.

**Table 2:** The characteristics of children and families in provincial units.

Provincial units	All children n (%)	Left-behind children n (%)	Migrant children n (%)	Parent’ agriculture hukou	Migrant distance		Province GDP (2014)	Household month income (USD) (Mean±SD)
					Across provinces	Within province		
Beijing	4477(3.8)	1423 (31.8)	3054 (68.2)	3104 (69.3)	4477 (100.0)	0.0	21330.8	1384.1 (1658.7)
Tianjin	4159(3.5)	1195 (28.7)	2964 (71.3)	3663 (88.1)	4159 (100.0)	0.0	15722.5	891.0 (741.6)
Hebei	2808 (2.4)	1020 (36.2)	1788 (63.7)	2488 (88.6)	1198 (42.7)	1610 (57.3)	29421.2	736.4 (538.2)
Shanxi	3092 (2.6)	618 (20.0)	2474 (80.0)	2652 (85.8)	1301 (42.1)	1791 (57.9)	12759.4	704.9 (650.5)
Neimenggu	3352 (2.9)	225 (6.7)	3127 (93.7)	2846 (84.9)	812 (24.2)	2540 (75.8)	17769.5	814.0 (716.2)
Liaoning	1964 (1.7)	421 (21.4)	1543 (78.6)	1607 (81.8)	1342 (68.3)	622 (31.7)	28626.6	865.6 (1059.6)
Jilin	1873 (1.6)	284 (15.2)	1589 (84.8)	1562 (83.4)	588 (31.4)	1285 (58.6)	13803.8	763.4 (967.7)
Heilongjiang	4643 (4.0)	538 (11.6)	4105 (88.4)	3716 (80.0)	827 (17.8)	3816 (82.2)	15039.4	728.9 (867.6)
Shanghai	4800 (4.1)	1927 (40.2)	2873 (59.8)	3783 (78.8)	4800 (100.0)	0.0	23560.9	1381.6 (2103.1)
Jiangsu	7310 (6.2)	2876 (39.3)	4434 (60.7)	6396 (87.5)	4947 (67.7)	2363 (32.3)	65088.3	1078.4 (1267.5)
Zhejiang	8435 (7.2)	4323 (51.3)	4112 (48.8)	7851 (93.1)	7577 (89.8)	858 (10.2)	40153.5	1068.2 (1439.0)
Anhui	3721 (3.2)	476 (12.8)	3245 (87.2)	3220 (86.4)	429 (11.5)	3292 (88.5)	20848.8	918.0 (707.8)
Fujian	4166 (3.6)	1538 (36.9)	2628 (63.1)	3877 (93.1)	2313 (55.5)	1853 (44.5)	24055.8	992.4 (1263.2)
Jiangxi	2940 (2.5)	1097 (37.3)	1843 (62.7)	2421 (82.4)	876 (29.8)	2064 (70.2)	15708.6	894.2 (1102.3)
Shandong	4395 (3.7)	802 (18.3)	3593 (81.8)	3942 (89.7)	586 (13.3)	3809 (86.7)	59426.6	893.4 (725.4)
Henan	3051 (2.6)	1088 (35.7)	1963 (64.3)	2880 (94.4)	608 (19.9)	2443 (80.1)	34939.4	779.4 (776.5)
Hubei	4166 (3.6)	612 (14.7)	3554 (85.3)	3702 (87.6)	1014 (24.3)	3152 (75.7)	29421.2	872.2 (997.4)
Hunan	3944 (3.4)	1428 (36.2)	2516 (63.8)	3415 (86.6)	499 (12.7)	3445 (87.4)	27048.5	865.3 (849.3)
Guangdong	7654 (6.5)	3018 (39.4)	4636 (60.6)	6706 (87.6)	5399 (70.5)	2255 (29.5)	67792.2	982.1 (1209.7)
Guangxi	3868 (2.7)	685 (17.7)	3183 (82.3)	3298 (85.3)	1043 (27.0)	2825 (73.0)	15673	764.3 (839.7)
Hainan	3187 (2.7)	564 (17.7)	2623 (82.3)	2537 (79.6)	1990 (62.4)	1197 (37.6)	3500.7	858.5 (860.6)
Chongqing	2710 (2.3)	970 (35.8)	1740 (64.2)	1858 (65.6)	846 (31.2)	1864 (68.8)	14265.4	915.7 (1081.4)
Sichuan	2918 (2.5)	1170 (40.1)	1748 (59.9)	2294 (78.6)	550 (18.9)	2368 (81.2)	28536.7	806.0 (998.5)
Guizhou	2212 (1.9)	518 (23.4)	1694 (76.6)	1929 (87.2)	719 (32.5)	1493 (67.5)	9251	878.9 (1018.9)
Yunan	2988 (2.5)	991 (33.2)	1997 (66.8)	2704 (90.5)	1453 (48.6)	1535 (51.4)	12814.6	853.8 (924.2)
Xizang	1254 (1.1)	821 (65.5)	433 (34.5)	1085 (86.5)	943 (72.5)	311 (24.8)	920.8	1012.3 (1572.9)
Shanxi	3869 (3.3)	1232 (31.8)	2637 (68.2)	3423 (88.5)	1314 (34.0)	2555 (66.0)	17659.4	770.6 (730.4)
Gansu	3450 (2.9)	981 (28.4)	2469 (71.6)	3071 (89.0)	1203 (34.9)	2247 (65.1)	6835.3	877.0 (1240.5)
Qinghai	2982 (2.5)	1217 (40.8)	1765 (59.2)	2685 (90.0)	1759 (59.0)	1223 (41.0)	2301.1	838.9 (1382.3)
Ningxia	2637 (2.2)	330 (12.5)	2307 (87.5)	2339 (88.7)	1131 (42.9)	1506 (57.1)	2752.1	807.3 (1234.2)
Xinjiang	2280 (1.9)	474 (20.8)	1806 (79.2)	1908 (83.7)	1600 (70.2)	680 (29.8)	9264.1	842.0 (912.4)
Xinjiang crops	2187 (1.9)	559 (25.6)	1628 (74.4)	1948 (88.2)	2145 (97.1)	64 (2.9)	-	735.5(1025.9)
Total	117492 (100.0)	35421 (30.1)	82071 (69.9)	100910 (85.8)	60473 (51.4)	57100 (48.6)	-	922.7(1150.1)

### Multilevel modeling analyses

#### Model 1

The results of multilevel modeling are shown in Table 3. Results of the null model showed that the unconditional mean of log-RR of LBC percentage is -1.33 (p < 0.01). It suggests that log-RR of LBC percentage vary over provinces and cities or districts, and the variance is 0.21 in log scale in provincial level (p < 0.0001) and 0.16 in log scale in cities or districts level (p < 0.0001).

**Table 3:** Three-level intercept random Poisson regression models for predicting related factors of children are left behind.

	Model 1		Model 2		Model 3	
Fix part	β (SE)	RR	β (SE)	RR	β (SE)	RR
Cons	-1.33(0.09)	0.26**	-3.16 (0.16)	0.04***	-3.06 (0.23)	0.05**
Girls (Ref: boys)			0.02 (0.01)	1.02†	0.02 (0.01)	1.02
Children’s age (Ref: aged 0–2 years)						
Children aged 3–5			0.12 (0.02)	1.02***	0.12 (0.02)	1.02***
Children aged 6–11			0.21 (0.02)	1.24***	0.21 (0.02)	1.24***
Children aged 12–14			0.40 (0.03)	1.49***	0.40 (0.03)	1.49***
Children aged 14–17			0.60 (0.03)	1.83***	0.60 (0.03)	1.83***
Two children (Ref: only child)			0.07 (0.1)	1.07***	0.07 (0.1)	1.07***
Three children and above			0.10 (0.3)	1.10***	0.10 (0.3)	1.10***
Parent’s agriculture hukou (Ref: non-agriculture)			-0.02 (0.02)	0.98	-0.02(0.02)	0.98
Parent’s education (Ref: college and above)						
Primary school or below			0.02(0.03)	1.02	0.02(0.03)	1.02
Middle school			0.04(0.03)	1.04	0.04(0.03)	1.04
High school			0.06(0.03)	1.06**	0.06(0.03)	1.06**
Parent’s age			-0.016 (0.001)	0.99**	-0.016 (0.001)	0.99**
Household monthly income (USD, Ref: > 1500)						
<300			0.13 (0.04)	1.14**	0.13 (0.04)	1.14**
300–750			0.11 (0.03)	1.11***	0.11 (0.03)	1.12***
750–1500			0.20 (0.03)	1.22***	0.19 (0.03)	1.21***
Two parents live separately (Ref: live together)			0.90 (0.01)	2.45***	0.89 (0.02)	2.44***
Willing to live in city (Ref: More than 5 years)						
Less than 5 years			0.42 (0.02)	1.51***	0.41 (0.02)	1.51***
No idea			0.33 (0.01)	1.39***	0.32 (0.01)	1.38***
Migrant across province (Ref: within province)			0.26 (0.01)	1.29***	0.26 (0.01)	1.29***
Housing ((Ref: own house)						
Rent employee offered apartment			0.94 (0.03)	2.56***	0.95 (0.03)	2.58***
Rent personal’s apartment			0.80 (0.03)	2.23***	0.80 (0.03)	2.23***
Rent government offered apartment			0.65 (0.11)	1.92***	0.66 (0.11)	1.93***
Employee offered free dormitory			0.68 (0.05)	1.97***	0.67 (0.06)	1.96***
Live in workplace			0.89 (0.04)	2.43***	0.88 (0.04)	2.42***
Live in unstable place			1.10 (0.03)	3.01***	1.10 (0.03)	3.01***
New rural cooperative medical insurance			-0.06 (0.02)	0.95**	-0.05 (0.02)	0.95**
Basic medical insurance for urban workers			0.03 (0.04)	1.03	0.04 (0.04)	1.04
Basic medical insurance for urban residents			0.00 (0.03)	1.00	-0.02 (0.04)	0.98
Cooperative medical insurance for Urban and Rural Residents			-0.11 (0.04)	0.90**	-0.10 (0.04)	0.90**
Maternity insurance			0.01 (0.03)	1.01	0.01 (0.03)	1.01
Unemployment insurance			0.05 (0.03)	1.05*	0.05(0.03)	1.05*
Old-age insurance for urban workers			0.04 (0.04)	1.04	0.04 (0.04)	1.04
Old-age insurance for urban residents			0.00 (0.03)	1.00	-0.01 (0.03)	0.99
Housing subsidies			-0.01 (0.03)	0.99	-0.01 (0.03)	1.00
New type of pension insurance for rural residents			0.04(0.02)	1.04*	0.04 (0.02)	1.04*
**Provincial level variables**						
Province average household monthly income					0.23 (0.11)	1.26†
Province GDP					0.02 (0.06)	1.02
Eastern areas (VS Northern areas)					0.17 (0.17)	1.19†
Middle areas (VS Northern areas)					0.17 (0.15)	1.18†
Western areas (VS Northern areas)					0.15 (0.05)	1.16†
**Random Part**	**β** (SE)	P	**β** (SE)	P	**β** (SE)	P
Level 3: province	0.07 (0.02)	<0.0001	0.06 (0.02)	0.001	0.04 (0.01)	<0.0001
Level 2: city/district	0.05 (0.006)	<0.0001	0.05 (0.006)	<0.0001	0.05 (0.006)	<0.0001
Units: province	32		32		31 ^a^	
Units: city/district	429		429		416	
Units: individual	117573		115859		113719	

^†^p < 0.10,

*p < 0.05,

**p < 0.01

***p < 0.0001;

^a.^ No GDP data for Xinjiang crops, so only 31 units were used. Parameter estimation (**β**/SE) is in log scale and RR value was generated by exponentiation value of **β**

#### Model 2

As expected that the variation at province and cities or districts levels decreased from null model to model 2 after explanatory individual level variables were added. The old children are more likely to be left behind than young children. Children aged 14–17 are most likely to be left behind (RR = 1.83, p < 0.0001). The risk for children aged 6–11 is 1.18 times (p < 0.0001). However, it should be noticed that children were grouped according to school system, so there were more children in both age group of 6–11 and 12–17, because it includes more school years than preschool. Children and parents’ agriculture hukou has no significant effect on children’s status (LBC or MC). Parents who only finished their middle and high school are more likely to leave their children in hometown comparing with parents who have college degrees (RR = 1.07, p < 0.01). Furthermore, high household monthly income is a protective factor for children been left behind. Parents who have the New Rural Cooperative Medical Insurance (RR = 0.95, p < 0.01), and Cooperative Medical Insurance (RR = 0.90, p < 0.01) are more likely to bring their children to the city. In contrast, parents who have unemployment insurance tend to leave their children behind (RR = 1.07, p < 0.01).

Parent migrated across the province are more likely to abandon their children in domicile place comparing within province floating (RR = 1.29, p < 0.0001). Parents living apart tend to have more left behind children (RR = 2.44, p < 0.0001). Parents’ long-term plan also plays an important role on children’s status. The result shows that parent intended to live in the city less than five years (RR = 1.51, p < 0.0001), or they have no plan (RR = 1.38, p < 0.0001) have a higher risk to leave their children in hometown than the parent who has determined to stay in the city over five years.

Moreover, the living condition is a crucial factor determining parent’s decisions. Parents who rent apartments provided to employees (RR = 2.58, p < 0.0001), personal apartment (RR = 2.23, p < 0.0001), and apartments provided by government (RR = 1.93, p < 0.0001) have a higher risk of leaving behind children compared to the group of own apartments. Also, parents who are living in free dormitories offered to employees (RR = 1.96, p < 0.0001), workplace (RR = 2.42, p < 0.0001) are more likely to leave their children behind. For children whose parents have no stable place to live have the highest risk (RR = 3.01, p < 0.0001) to be left behind.

#### Model 3

High average household monthly income in the destination province is predictive of more left behind children (model 3). Increasing one unit (in log scale) of the average monthly income of province will increase 1.98 times risk to leave their children behind (p < 0.1). Also, parents working in western regions are 1.41 times as likely as parents working in northern areas to abandon their children (RR = 1.41, p < 0.1). Similarly, compared with northern cities, parents migrated to eastern cities have a risk of 1.40 times to leave their children behind, followed by middle regions (RR = 1.38, p < 0. 1). Province GDP shows no significant effect on children’s status. Moreover, the variance attributable to cities is sustainable in three models, even after taking the detailed information on households and provinces into consideration.

## Discussion

The number of migrant families with young children has been increased significantly in China over the last decade. While more and more migrant parents decided to bring their children with them to urban areas, there are still some structural and social barriers that would discourage children from moving to cities with their parents. The current study found several migration-related factors including housing condition at destination, migrant distance, and the geographical location of host city that would strongly predict parents’ decision to leave their children behind.

First, the housing conditions of migrants in host cities are crucial factor determining the mobility of children with their parents, which is consistent with the findings in previous study [31]. Previous studies have repeatedly documented a poor living condition of migrants in destinations [32–34] because they are marginalized by the urban housing system. A national survey indicated that the average living area size for migrant families is 30–60 square meters, while 19.3% households live in spaces between 10–30 square meters. The previous study also suggested that over one half of MC lacked a study room in their urban dwellings [1]. The findings of the current study suggested a relatively high risk for each type of housing conditions in predicting LBC based on quantitative analysis, although the common perception was that only parents who lived in employee-offered free dormitories were more likely to leave their children behind because most of these facilities consisted of one large room shared by multiple people [27].

Second, migration distance is associated with a family’s decision to leave their children behind. The current study found that parents moved across provinces are more likely to leave their children behind than the parents who migrated within provinces. Parents planed for short term work in cities or without a plan of longer stay preferred to leave their children behind.

Third, the geographical locations of migration destination are also related to the distribution of LBC and MC. We found that parents migrating to the eastern areas are more likely to leave their children behind, followed by the western areas. This pattern reflects the levels of socioeconomic developments of these two areas, although for different reasons. For the eastern coastal areas which are more economically developed, the cost of living including both housing and schooling may be substantially higher than other areas in the country, so migrant parents might decide to leave their children behind because of the limited affordability of raising their children in these areas. On the contrary, for the mid-western areas which are less economically developed in the country, the social infrastructure such as quality of schools may not be as good as other areas, so parents migrating to these areas may decide to leave their children in their origin because of the potentially low return on their investments on children’s schooling. An interesting fact is that most LBC are also living in mid-western provinces and economically developed eastern provinces in China according to the population census in 2010 [1]. Both phenomena may reflect a combination of migratory labor input and output in these areas at the two ends of economic development spectrum in China.

One of family level factors that may explain the differences in the distribution of LBC is the migrant household family income at the destination which was positively related to the family’s decision to leave their children behind. One possible explanation for this finding is that these migrant parents might choose to do so to avoid the conflicts between working long hours (to maximize income) and raising children. A previous study found that the average working hours for migrant workers is 63.1 hours per week [35] and working hours are significantly related with income [36]. However, we did not find a significant relationship between provincial GDP and the number of LBC after control for migrant household family income and other factors. Besides, we found that the destination cities sustainably contributed the variance on proportion of LBCs. It’s probably because migrants usually prefer provincial capitals or well-developed cities to other cities within a province.

At individual level, children’s age seemly affect parent’s decision to leave their children behind. Adolescent children are more likely to be left behind than younger ones, but preschoolers are also more likely to be left behind than their older siblings. One possible reason is that older children, especially children aged 14–17, are more likely able to take care themselves if they are left behind. Sometimes adolescents are left behind to take care of the remaining family, including their younger siblings [37]. Another reason for older children being left behind is the schooling issues of these children as the China National Higher Education Entrance Examination prohibits students from taking the examination in urban areas if they do not have a local hukou (so they must return to their hometown to take the examination even if they migrated to cities with their parents). Schooling may be also the reason for preschool-aged children being left behind. The number of preschool-age LBC in China has been increased to 23.42 million in 2010 based on the 2010 National Population Census [1], representing an increase of 47.7% in comparison to 2005. There may be limited schooling opportunities for these children in urban areas as the preschool education is not part of the 9-year compulsory education system in China and there are very few public preschools in urban areas. Migrant parents would find it difficult to afford the private preschools which are often expensive.

### Implications of the findings

Earlier parental deprivation among rural left behind children was found to be related to worse child outcomes, especially for children less than 3 years of age [38], which is consistent with other literature on the impact of early separation from caregivers and subsequent functioning [39,40]. Considering the early parental separation is the primary cause for various behavioral and developmental problems of LBC [41], encouraging the reunification of parents and children probably is the best way to address the problems of LBC. Leaving one parent at home or bringing children into the cities are the two possible ways to address the problem of parental deprivation. More migrant families are opting for the latter choice, and this trend seems set to continue. The findings of the current study may suggest some directions for the government and society in the destination areas to assist and support migrant parents to bring their children with them to the urban area.

First, destination cities should strengthen their capability to accommodate migrant families with children, especially by improving housing conditions. A dominant view in the public media and endorsed by the central government is that the state should directly provide affordable housing for low income households including migrant workers. The China’s 12th national ‘Five Year Plan’ introduced public rental houses that could benefit migrant families to some extent [42]. However, research also suggests other feasible housing options for migrant workers. Because the lowest income workers and newcomers tended to live in employer-provided accommodation [43], one option would require employers, especially factories located in suburbs, to provide affordable accommodations for migrant families.

Second, the destination community should provide or increase schooling opportunities for migrant children. The development of migrant children could be safe-guarded by enhancing their opportunity to enroll in public schools and by improving the quality of the migrant schools. The former strategy may be more advantageous than the latter because it allows for the social integration of rural migrant children within the school context. Preschoolers and adolescent children are two groups at particularly high risk of being left behind. Therefore, a possible solution is to improve the kindergarten enrollment rate of migrant children by opening more public kindergartens or establishing new special and affordable preschools in cities for migrant children. Reform of the national college entrance examination system would be a further strategy to reduce the prevalence of adolescent children being left behind in rural areas.

There are existing program of interventions targeting LBC (as well as MC) to improve their living circumstance and developmental outcomes, but many gaps remain [44–46]. One of the gaps in the existing efforts is that almost all of the previous studies targeted only LBC or MC, which may represent a missed opportunities or misdirection of resources [47] as the LBC and MC could actually represent different stages of a continuum of the children migration. The national data suggested an average migration age of 4.5 years for children aged 8 to 10 [1]. Therefore, future efforts need to develop a comprehensive strategy to support the entire family of migrants which include parents, LBC and MC to mitigate any negative effects of family migration on the children development and to capitalize the potential benefits of family migration to the physical, social, and psychological wellbeing of migrant children.

## Strengths and limitations

There are several strengths of this study including national representative data with a large sample size and multilevel analysis to deal with three-level data. In addition, information at migration destination (e.g., GDP, geographic location) was taken into consideration. The limitations of the study included the limited variables available from the national survey, especially, the lack of information on labor output areas, which prevented us from showing a comprehensive picture of the complex social factors. The survey collection may be subject to self-report bias in some variables. For example, the first child parents filled in the table was selected as the index child in families with the two and above children would results in selection bias.
